# Peer bullying victimisation and depressive symptoms as serial mediators between attention‐deficit/hyperactivity disorder symptoms and internet gaming disorder among Chinese adolescents: A three‐wave longitudinal study

**DOI:** 10.1002/gps3.70012

**Published:** 2026-03-23

**Authors:** Pu Peng, Zhangming Chen, Silan Ren, Ying He, Jinguang Li, Aijun Liao, Linlin Zhao, Xu Shao, Shanshan Chen, Ruini He, Yudiao Liang, Youguo Tan, Xiaogang Chen, Jinsong Tang, Yanhui Liao

**Affiliations:** ^1^ Department of Psychiatry Sir Run Run Shaw Hospital Zhejiang University School of Medicine Hangzhou Zhejiang China; ^2^ Department of Psychiatry Zigong Mental Health Center Zigong Sichuan China; ^3^ Department of Nursing Sichuan Vocational College of Health and Rehabilitation Zigong Sichuan China; ^4^ Department of Psychiatry National Clinical Research Center for Mental Disorders, and National Center for Mental Disorders The Second Xiangya Hospital of Central South University Changsha Hunan China

**Keywords:** adolescent, attention‐deficit/hyperactivity disorder, depression, internet gaming disorder, peer bullying

## Abstract

**Background:**

The association between attention‐deficit/hyperactivity disorder (ADHD) symptoms and internet gaming disorder (IGD) is well‐established, yet the psychological mechanisms underlying this comorbidity remain underexplored.

**Aims:**

Grounded in the dual failure model and the compensatory internet use model, this study examined peer bullying victimisation and depressive symptoms as serial mediators in the longitudinal association between ADHD symptoms and IGD severity among 20 137 Chinese adolescents.

**Methods:**

Participants were assessed at baseline (T1, November 2020) and followed up at one (T2) and two years (T3). Standardised measures assessed peer bullying victimisation (Multidimensional Peer Victimisation Scale), ADHD symptoms (Strengths and Difficulties Questionnaire), depressive symptoms (9‐item Patient Health Questionnaire) and IGD severity (Internet Gaming Disorder Scale–Short Form). Longitudinal path analysis with serial mediation tested the hypothesised pathway, adjusting for baseline covariates and prior symptoms. Subgroup analyses examined sex and developmental (early vs. late adolescence) differences. Sensitivity analyses included alternative mediation models, cross‐lagged panel models and parallel‐process latent growth curve models.

**Results:**

Baseline ADHD symptoms directly predicted IGD severity and indirectly through peer bullying victimisation and depressive symptoms. These mediators accounted for one‐third of the total effect. The bullying‐related mediation pathway was evident only among boys and early adolescents, whereas depressive symptoms consistently mediated the association across sexes and age groups. Sensitivity analyses supported the robustness and temporal specificity of the proposed pathway.

**Conclusions:**

ADHD symptoms increase the risk of subsequent IGD through both direct and indirect pathways operating through peer bullying victimisation and depressive symptoms. This social–emotional mediation process is developmentally and sex contingent. These findings suggest that effective prevention and intervention for IGD in adolescents with ADHD should incorporate developmentally and sex‐sensitive strategies that address peer victimisation and emotional distress in addition to core ADHD symptoms.

## Abstract (highlights)

**WHAT IS ALREADY KNOWN ON THIS TOPIC**

- Attention‐deficit/hyperactivity disorder (ADHD) symptoms and internet gaming disorder (IGD) are highly comorbid in adolescents and linked to negative outcomes, yet the psychological mechanisms underlying this comorbidity remain underexplored.

**WHAT THIS STUDY ADDS**

- Grounded in the dual failure model and the compensatory internet use model, this large longitudinal study is the first to demonstrate that peer bullying victimisation and depressive symptoms sequentially mediate the relationship between ADHD symptoms and later IGD severity. The bullying‐related pathway is specific to boys and early adolescence, whereas depressive symptoms act as a more pervasive mediator.

**HOW THIS STUDY MIGHT AFFECT RESEARCH, PRACTICE OR POLICY**

- These findings highlight critical interpersonal and emotional mechanisms underlying the ADHD‐IGD link, providing empirical support for theoretical models. Clinically, the results underscore the importance of assessing and targeting peer victimisation, depressive symptoms and ADHD symptoms in interventions for IGD, suggesting the need for tailored approaches based on sex and developmental stages.

## INTRODUCTION

Attention‐deficit/hyperactivity disorder (ADHD) and internet gaming disorder (IGD) are significant public health concerns among adolescents globally, frequently exhibiting considerable comorbidity.^1^ Both conditions are linked to impaired academic performance, social difficulties and psychological distress.^2^, ^3^, ^4^

The relationship between ADHD symptoms and IGD has attracted increasing research attention due to their significant clinical overlap. A recent meta‐analysis of cross‐sectional studies revealed a moderate positive association (*r* = 0.296) between ADHD symptom severity and IGD severity.^5^ Longitudinal research studies, primarily in community samples, further indicate that ADHD symptoms are a significant prospective predictor of both the onset and persistence of IGD,^6^ although findings are not universally consistent.^7^, ^8^ In clinical populations diagnosed with IGD, comorbid ADHD is frequently observed and is associated with a more severe clinical presentation, a longer illness trajectory and lower recovery rates.^9^, ^10^ Furthermore, preliminary studies suggest that pharmacological interventions targeting ADHD symptoms, such as methylphenidate, may hold promise in reducing IGD severity.^11^, ^12^ Collectively, these findings underscore the significant and complex role of ADHD symptoms in the development, maintenance and potential treatment response of IGD.

Despite this growing body of evidence, the specific psychological and interpersonal mechanisms that explain how ADHD symptoms contribute to the development of maladaptive gaming behaviours remain poorly understood. Identifying these mediating pathways is critical for developing targeted prevention and intervention strategies. To date, only one longitudinal study has attempted to test a potential mediating process in this relationship.^13^ This study of 1732 Korean adolescents identified a serial mediation pathway from low self‐control to aggression, linking ADHD symptoms to IGD. Although valuable, the authors noted that the effect sizes for this specific pathway were relatively small, suggesting the likely involvement of other mediators.

Integrating theoretical perspectives such as the dual failure model and the compensatory internet use model offers a compelling framework for conceptualising alternative mediating processes. The dual failure model posits that disruptive externalising behaviours characteristic of ADHD symptoms can lead to chronic negative peer interactions, including peer rejection and bullying victimisation—a form of ‘social failure’.^14^, ^15^ Experiencing such persistent social adversity is theorised to subsequently trigger or exacerbate internalising symptoms, particularly depression. The compensatory internet use model extends this by suggesting that depressive symptoms drive compensatory gaming behaviours, where individuals use gaming as a maladaptive coping strategy to regulate negative emotions and escape real‐world stressors.^16^ These models together predict a sequential mediation pathway: ADHD symptoms → bullying victimisation → depressive symptoms → IGD.

Beyond psychosocial models, growing evidence suggests that ADHD symptoms, sensitivity to social stressors such as peer bullying, depressive symptoms and addictive behaviours, including IGD, may also share common neurobiological vulnerabilities. These include networks involved in cognitive control (e.g., anterior cingulate and dorsolateral prefrontal cortices), reward valuation and motivation (e.g., striatal and orbitofrontal regions) and affective processing and regulation (e.g., insular and amygdala systems).^17^, ^18^, ^19^, ^20^, ^21^ Alterations in these systems have been linked to attentional dysregulation, heightened rejection sensitivity, maladaptive self‐referential processing and compensatory engagement in rewarding digital activities. From this perspective, psychosocial stressors and emotional symptoms may interact with underlying neural vulnerabilities, jointly shaping developmental pathways towards problematic gaming behaviours during adolescence.

Despite this theoretical support, empirical validation of this integrated mechanism is limited. Although cross‐sectional studies have explored isolated mediators (e.g., depression and hopelessness accounting for a significant portion of the ADHD‐IGD relationship),^22^ cross‐sectional designs cannot establish temporal precedence or test complex serial mediation processes. Notably, no longitudinal study has yet rigorously tested the specific serial mediating effect of peer bullying victimisation followed by depressive symptoms in the relationship between ADHD symptoms and IGD.

To address these gaps, we conducted a large‐scale longitudinal study among 20 137 Chinese adolescents over a 2‐year period. The primary objectives were as follows: (1) to examine the prospective association between baseline ADHD symptoms and the development/severity of IGD over time and (2) to rigorously test the hypothesised serial mediation pathway (ADHD symptoms → peer bullying victimisation → depressive symptoms → IGD) within this longitudinal framework. Given the established sex differences in IGD prevalence and clinical presentation,^23^ and previous research suggesting that the ADHD‐IGD relationship or its mediating pathways may vary by sex,^13^ we performed subgroup analyses to explore potential sex‐specific effects in the proposed serial mediation model.

## METHODS

### Study procedure and participants

**FIGURE 1:**
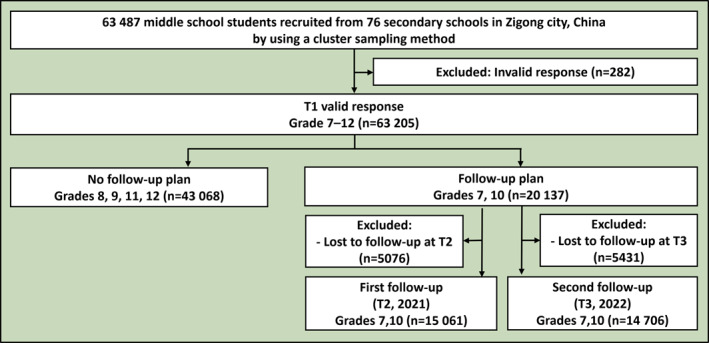
Study flowchart.

This study utilised data from a three‐wave longitudinal cohort of adolescents in Zigong City, China. The detailed study protocol and part of the data have been published previously.^24^, ^25^, ^26^, ^27^, ^28^, ^29^ As shown in figure 1, an initial cohort of 63 487 students (Grades 7–12) was recruited through a cluster sampling method from 76 schools in November 2020 (T1). Among them, 63 205 provided valid responses. For follow‐ups in November 2021 (T2) and 2022 (T3), the sample was prospectively focused on students who were in Grades 7 and 10 at baseline (*n* = 20 137).

To ensure standardised administration, all teachers involved in supervising the data collection received protocol training prior to each wave. Participants completed the psychological assessments on computers in designated school laboratories during scheduled sessions. These sessions were overseen by the trained teachers, who were available to answer questions. After the data were gathered, a stringent quality assurance procedure was applied. Any submitted surveys were removed from the final dataset if they demonstrated (1) an exceptionally brief completion time suggesting nonengagement, (2) logically inconsistent answers such as an impossible age (e.g., 999) or (3) demographic data that was incongruent across the study's waves.

The study protocol was reviewed and approved by the Ethics Committee of the Zigong Mental Health Center (Approval No. 2020‐8‐01). Written informed consent was obtained from all participants and guardians of minors, with full disclosure of study objectives.

### Measurements

#### Predictor (ADHD symptoms)

ADHD symptoms were assessed using the 5‐item subscale of the Strengths and Difficulties Questionnaire (SDQ), a widely validated behavioural screening tool.^30^, ^31^ This subscale measures symptom frequency over the preceding 6 months. The 5 items include three assessing hyperactivity/impulsivity and two assessing inattention.^32^ Items were rated on a 3‐point scale (0 = not true, 1 = somewhat true, 2 = certainly true). A total score ranging from 0 to 10 was calculated by summing the item scores, with reverse‐scored items appropriately handled. Scores of ≥ 7 are often considered indicative of clinically significant symptoms.^33^ The Chinese version of the SDQ has demonstrated acceptable internal consistency and established discriminant validity in adolescent populations.^31^

#### Mediators (peer bullying and depressive symptoms)

Peer victimisation was measured using the Multidimensional Peer Victimisation Scale (MPVS), a validated tool assessing the frequency of traditional school bullying over the past 6 months.^34^ Participants rated each item on a 3‐point Likert scale (0 = not at all; 1 = once; 2 = more than once), with higher scores indicating greater victimisation. The Chinese version of the MPVS has displayed excellent psychometric properties,^35^ with high internal consistency in the present sample (Cronbach's *α* > 0.85 across three waves). The MPVS score was quartiled to reflect no, mild, moderate and severe bullying victimisation.

Depressive symptoms were assessed using the 9‐item Patient Health Questionnaire (PHQ‐9). This instrument evaluates depressive symptoms over a 2‐week recall period on a 4‐point Likert scale (0 = not at all; 3 = nearly every day), with scores ranging from 0 to 27. Scores ≥ 10 indicate clinically significant depression.^36^ The Chinese version of the PHQ‐9 demonstrated strong psychometric properties and has been widely used in the Chinese population,^37^, ^38^, ^39^ with high internal consistency in our cohort (*α* > 0.9 across three waves).

#### Outcome (IGD)

IGD symptoms were measured using the 9‐item Internet Gaming Disorder Scale–Short Form (IGDS9‐SF) across all three waves (T1–T3). The scale assesses Diagnostic and Statistical Manual of Mental Disorders (Fifth Edition) diagnostic criteria for IGD over the past 12 months,^40^, ^41^, ^42^ with participants rating symptom frequency on a 5‐point Likert scale (1 = never; 5 = very often). A composite severity score ranging from 9 to 45 was generated. A score of ≥ 32 was used to define clinically significant IGD.^43^ The IGDS9‐SF demonstrated excellent internal consistency across waves (T1: *α* = 0.91; T2: *α* = 0.91; T3: *α* = 0.92).

#### Baseline demographic covariates

Demographic control variables included sex (male/female), age, residential classification (urban/rural) and academic grade. Family characteristics were categorised as follows: only‐child status (yes/no), left‐behind status (parental migration history) and family structure (nuclear/single‐parent or remarried). Recent substance use behaviours (tobacco and alcohol consumption in the past 30 days) and sleep duration were also included as behavioural covariates.

To measure gaming engagement, participants reported their average daily gaming duration (hours) during weekdays and weekends over the past year. Weekly total gaming exposure was calculated using the following formula: (mean weekday hours × 5) + (mean weekend hours × 2).

### Statistical analysis

Descriptive statistics were used to summarise sample characteristics, with continuous variables presented as means (standard deviations [SDs]) and categorical variables as frequencies (percentages). Baseline characteristics between adolescents with and without IGD were compared using independent‐samples *t* tests or chi‐square tests, as appropriate. Logistic regression models were first applied to examine associations among hyperactivity/inattention, peer bullying victimisation, depressive symptoms and IGD, adjusting for relevant covariates. Baseline IGD severity was controlled for in all longitudinal analyses.

#### Primary longitudinal mediation analysis

The primary hypothesis was tested using a three‐wave longitudinal path analysis within a structural equation modelling framework. Full information maximum likelihood estimation was employed to address missing data. This mediation model specified hyperactivity/inattention at T1 (measured by the SDQ subscale) as the predictor, peer bullying victimisation (MPVS) and depressive symptoms (PHQ‐9) at T2 as sequential mediators and IGD severity at T3 (IGDS9‐SF) as the outcome. To account for prior symptom levels and reduce potential confounding by temporal stability, T2 mediators were adjusted for their corresponding baseline levels and T3 IGD was adjusted for baseline IGD severity. All models additionally controlled for baseline covariates, including demographic characteristics and other relevant psychosocial factors. This three‐wave longitudinal mediation approach has been widely applied in prior research on psychiatry to examine temporally ordered mediation processes.^44^, ^45^ Indirect effects were estimated via bias‐corrected bootstrapping (5000 resamples; 95% confidence intervals [CIs]). Because the primary longitudinal mediation model was just identified, global model fit indices were not interpreted.

#### Sex and developmental differences in the mediation framework

To examine potential heterogeneity in the proposed mediation pathways, multigroup analyses were conducted by sex (male vs. female) and developmental stage (Grade 7 vs. Grade 10, representing early and late adolescence, respectively). We tested for moderation by examining measurement invariance and then sequentially constraining critical pathways (T1 ADHD symptoms → T2 bullying, T2 bullying → T2 depression, T2 depression → T3 IGD, T1 ADHD symptoms → T3 IGD, T2 bullying → T3 IGD) to be equal across sexes. Nested model comparisons using likelihood ratio tests were used to determine if constraining the paths significantly worsened model fit, indicating a statistically significant difference in the pathway strength between the subgroups.

#### Sensitivity analyses

A series of sensitivity analyses was conducted to evaluate the robustness and specificity of the primary findings.

First, alternative mediation structures were examined, including a reversed serial mediation model in which depressive symptoms preceded peer bullying victimisation (i.e., ADHD symptoms → depressive symptoms → bullying victimisation → IGD), as well as a parallel mediation model in which peer bullying and depressive symptoms were specified as independent mediators. These models were compared with the primary model based on key path coefficients, indirect effects and explained variance rather than global fit indices.

Second, to explicitly account for autoregressive stability and examine potential reciprocal associations, cross‐lagged panel models (CLPMs) were estimated. ADHD symptoms, peer bullying victimisation, depressive symptoms and IGD were specified at all three waves, with autoregressive paths included for each construct. Baseline covariates were included as predictors of the observed variables at each wave. Model fit was evaluated using multiple indices, including the comparative fit index (CFI), root mean square error of approximation (RMSEA) and standardised root mean square residual (SRMR). Acceptable model fit was defined as CFI ≥ 0.90 and RMSEA and SRMR ≤ 0.08. Given the three‐wave design, the CLPM was not intended to provide a definitive test of a fully time‐separated serial mediation chain, which would require at least four waves. Instead, this analysis was designed to (i) examine whether ADHD symptoms prospectively predicted later IGD severity after accounting for autoregressive stability and reciprocal effects and (ii) explore whether key components of the hypothesised social–emotional pathway (bullying and depressive symptoms) were observable within a conservative cross‐lagged framework.

Third, to characterise shared developmental trajectories over time, a parallel‐process latent growth curve model was conducted. Intercepts and linear slopes were specified for ADHD symptoms, peer bullying victimisation, depressive symptoms and IGD severity across the three waves. Covariances among intercepts and among slopes were examined to assess co‐developmental associations across the study period. Baseline covariates were included as predictors of both intercept and slope factors. Model fit was evaluated using CFI, RMSEA and SRMR, with the same cut‐off criteria applied.

All analyses were conducted using R 4.2.0 for descriptive statistics and regression models, and Mplus 8.3 for mediation and path analyses. The significance threshold was set at *p* < 0.05 (two‐tailed).

## RESULTS

### Sample characteristics

At baseline (T1), the study enrolled 20 137 adolescents. Follow‐up assessments were conducted at T2 and T3, with 15 061 participants retained at T2 (74.79% retention) and 14 706 at T3 (73.03% retention). Overall, 16 982 participants (84.33%) completed at least one follow‐up assessment, and 12 785 (63.49%) completed all three waves. Attrition analysis indicated no significant differences in primary study variables, including IGD status (*χ* ^2^ = 1.59, *p* = 0.451), hyperactivity/inattention (*χ* ^2^ = 1.58, *p* = 0.454), peer bullying victimisation (MPVS; *F* = 2.37, *p* = 0.094) and depressive symptoms (*χ* ^2^ = 4.31, *p* = 0.116) (table S1). However, participants retained across waves differed on several demographic and background characteristics, including age (*F* = 11.15, *p* < 0.001), sex (*χ* ^2^ = 18.09, *p* < 0.001), residence (*χ* ^2^ = 36.24, *p* < 0.001), only‐child status (*χ* ^2^ = 21.69, *p* < 0.001), father's education level (*χ* ^2^ = 13.00, *p* = 0.002), mother's education level (*χ* ^2^ = 20.15, *p* < 0.001), grade (*χ* ^2^ = 28.18, *p* < 0.001) and sleep duration (*F* = 3.66, *p* = 0.026).

Baseline sample characteristics are detailed in table 1. The mean age was 13.40 years (SD = 1.45), with a balanced sex distribution (49.42% male). The majority of participants resided in rural areas (64.06%). Analysis of family structure showed that 77.50% were not only children, 33.54% were left‐behind children and 79.34% lived in nuclear families. Parental education levels were relatively low, with 76.74% of fathers and 79.61% of mothers having an education level below high school.

**TABLE 1:** Sample characteristics between adolescents with and without IGD

Variables	Total (*n* = 20 137)	Without IGD (*n* = 19 621)	With IGD (*n* = 516)	Statistic	*p* ^a^
Age, mean (SD)	13.40 (1.45)	13.40 (1.45)	13.51 (1.38)	*t* = −1.68	0.092
Gender, *n* (%)				*χ* ^2^ = 63.01	**< 0.001**
Boys	9952 (49.42)	9608 (48.97)	344 (66.67)		
Girls	10 185 (50.58)	10 013 (51.03)	172 (33.33)		
Grade, *n* (%)				*χ* ^2^ = 0.26	0.612
7	13 361 (66.35)	13 024 (66.38)	337 (65.31)		
10	6776 (33.65)	6597 (33.62)	179 (34.69)		
Residence, *n* (%)				*χ* ^2^ = 0.00	0.959
Country	12 900 (64.06)	12 570 (64.06)	330 (63.95)		
Urban	7237 (35.94)	7051 (35.94)	186 (36.05)		
Only child, *n* (%)				*χ* ^2^ = 8.88	0.003
No	15 606 (77.50)	15 234 (77.64)	372 (72.09)		
Yes	4531 (22.50)	4387 (22.36)	144 (27.91)		
Left‐behind child, *n* (%)				*χ* ^2^ = 8.54	0.003
No	13 383 (66.46)	13 071 (66.62)	312 (60.47)		
Yes	6754 (33.54)	6550 (33.38)	204 (39.53)		
Father education level, *n* (%)				*χ* ^2^ = 0.40	0.525
Below high school	15 453 (76.74)	15 051 (76.71)	402 (77.91)		
High school or above	4684 (23.26)	4570 (23.29)	114 (22.09)		
Mother education level, *n* (%)				*χ* ^2^ = 0.28	0.594
Below high school	16 032 (79.61)	15 626 (79.64)	406 (78.68)		
High school or above	4105 (20.39)	3995 (20.36)	110 (21.32)		
Family type, *n* (%)				*χ* ^2^ = 21.82	**< 0.001**
Nuclear family	15 977 (79.34)	15 610 (79.56)	367 (71.12)		
Single parent or remarried	4160 (20.66)	4011 (20.44)	149 (28.88)		
Alcohol use, *n* (%)				*χ* ^2^ = 197.59	**< 0.001**
No	16 742 (83.14)	16 431 (83.74)	311 (60.27)		
Yes	3395 (16.86)	3190 (16.26)	205 (39.73)		
Smoking, *n* (%)				*χ* ^2^ = 304.25	**< 0.001**
No	18 743 (93.08)	18 362 (93.58)	381 (73.84)		
Yes	1394 (6.92)	1259 (6.42)	135 (26.16)		
Gaming time, mean (SD)	11.20 (19.31)	10.50 (17.86)	38.01 (41.06)	*t* = −15.18	**< 0.001**
Sleep duration, mean (SD)	7.12 (1.46)	7.14 (1.44)	6.32 (1.72)	*t* = 10.84	**< 0.001**
Depressive symptoms, *n* (%)				*χ* ^2^ = 368.45	**< 0.001**
No	15 820 (78.56)	15 633 (79.67)	187 (36.24)		
Yes	4317 (21.44)	3988 (20.33)	329 (63.76)		
Hyperactivity/inattention, *n* (%)				*χ* ^2^ = 563.18	**< 0.001**
No	18 155 (90.16)	17 818 (90.81)	337 (65.31)		
Yes	1982 (9.84)	1803 (9.19)	179 (34.69)		
Peer bullying, *n* (%)				*χ* ^2^ = 495.35	**< 0.001**
No	5346 (26.55)	5307 (27.05)	39 (7.56)		
Mild	4736 (23.52)	4692 (23.91)	44 (8.53)		
Moderate	5388 (26.76)	5281 (26.92)	107 (20.74)		
Severe	4667 (23.18)	4341 (22.12)	326 (63.18)		

*Note*: Bold suggests statistical significance.

Abbreviations: ADHD, attention‐deficit/hyperactivity disorder; IGD, internet gaming disorder; SD, standard deviation.

^a^ For *t*‐tests or Chi‐square tests.

With respect to behavioural characteristics, 16.86% of participants reported alcohol use, and 6.92% reported smoking. The average weekly gaming time was 11.20 h (SD = 19.31) and the mean nightly sleep duration was 7.12 h (SD = 1.46). Based on established clinical cutoffs, 21.44% of adolescents exhibited clinically significant depressive symptoms, 9.84% showed elevated hyperactivity/inattention symptoms and 73.45% reported experiencing peer bullying victimisation of any severity.

### Cross‐sectional and longitudinal association of ADHD symptoms, depressive symptoms and peer bullying victimisation with IGD across three waves

The prevalence of IGD was 2.56% at T1, 1.48% at T2 and 1.41% at T3. At baseline, adolescents with IGD showed substantially higher rates of clinically significant hyperactivity/inattention symptoms compared with those without IGD (34.69% vs. 9.19%, *χ* ^2^ = 563.18, *p* < 0.001). Similarly, depressive symptoms were more prevalent among adolescents with IGD than among their non‐IGD peers (63.76% vs. 20.33%, *χ* ^2^ = 368.45, *p* < 0.001). Severe peer bullying victimisation was also markedly more common in the IGD group (63.18%) compared with those without IGD (22.12%, *χ* ^2^ = 495.35, *p* < 0.001).

Regression analyses (table 2) demonstrated strong cross‐sectional associations at T1 between ADHD symptoms, depressive symptoms, peer bullying victimisation and IGD. In unadjusted models, T1 ADHD symptoms were associated with higher odds of T1 IGD (odds ratio [OR] = 5.25, 95% CI 4.35–6.33, *p* < 0.001), as were T1 depressive symptoms (OR = 6.90, 95% CI 5.74–8.28, *p* < 0.001). Compared with no bullying, moderate bullying (OR = 2.76, 95% CI 1.91–3.99, *p* < 0.001) and severe bullying (OR = 10.22, 95% CI 7.31–14.28, *p* < 0.001) were associated with substantially elevated odds of T1 IGD. After adjustment for baseline covariates, these associations attenuated but remained statistically significant at T1: ADHD symptoms (adjusted OR [aOR] = 2.21, 95% CI 1.78–2.74, *p* < 0.001), depressive symptoms (aOR = 2.78, 95% CI 2.24–3.46, *p* < 0.001), moderate bullying (aOR = 1.86, 95% CI 1.26–2.73, *p* = 0.002) and severe bullying (aOR = 3.97, 95% CI 2.76–5.71, *p* < 0.001).

**TABLE 2:** Association of hyperactivity/inattention, depressive symptoms and peer bullying victimisation with IGD

Characteristics	IGD at T1		IGD at T2		IGD at T3	
	Unadjusted model	Adjusted model^a^	Unadjusted model	Adjusted model^b^	Unadjusted model	Adjusted model^b^
Hyperactivity/inattention	5.25 (4.35–6.33)***	2.21 (1.78–2.74)***	3.95 (2.96–5.26)***	1.49 (1.07–2.09)*	3.60 (2.65–4.90)***	1.88 (1.32–2.67)***
Depressive symptoms	6.90 (5.74–8.28)***	2.78 (2.24–3.46)***	5.36 (4.12–6.97)***	2.68 (1.93–3.72)***	3.37 (2.57–4.43)***	1.78 (1.28–2.50)***
Peer bullying victimisation						
No	1.00 (reference)	1.00 (reference)	1.00 (reference)	1.00 (reference)	1.00 (reference)	1.00 (reference)
Mild	1.28 (0.83–1.97)	1.15 (0.74–1.80)	1.71 (1.01–2.89)*	1.56 (0.92–2.66)	1.22 (0.71–2.07)	1.22 (0.71–2.08)
Moderate	2.76 (1.91–3.99)***	1.86 (1.26–2.73)**	2.94 (1.83–4.73)***	1.95 (1.19–3.19)**	2.30 (1.45–3.65)***	1.91 (1.19–3.08)**
Severe	10.22 (7.31–14.28)***	3.97 (2.76–5.71)***	5.59 (3.55–8.80)***	1.69 (1.02–2.80)*	4.48 (2.90–6.92)***	2.29 (1.42–3.69)***

Abbreviation: IGD, internet gaming disorder.

^a^ Adjusted for baseline covariates.

^b^ Adjusted for baseline covariates and IGD status.

*p < 0.05, **p < 0.01, ***p < 0.001.

In longitudinal models predicting later IGD, baseline ADHD symptoms and depressive symptoms remained significant predictors of T2 IGD after full adjustment (ADHD symptoms: aOR = 1.49, 95% CI 1.07–2.09, *p* = 0.021; depressive symptoms: aOR = 2.68, 95% CI 1.93–3.72, *p* < 0.001). For bullying, moderate victimisation predicted higher odds of T2 IGD (aOR = 1.95, 95% CI 1.19–3.19, *p* = 0.009), as did severe victimisation (aOR = 1.69, 95% CI 1.02–2.80, *p* = 0.042), whereas mild victimisation was not statistically significant (aOR = 1.56, 95% CI 0.92–2.66, *p* = 0.106).

Similarly, baseline ADHD symptoms and depressive symptoms significantly predicted T3 IGD (ADHD symptoms: aOR = 1.88, 95% CI 1.32–2.67, *p* < 0.001; depressive symptoms: aOR = 1.78, 95% CI 1.28–2.50, *p* < 0.001). Moderate (aOR = 1.91, 95% CI 1.19–3.08, *p* = 0.007) and severe bullying (aOR = 2.29, 95% CI 1.42–3.69, *p* < 0.001) were also significant predictors of T3 IGD, whereas mild bullying was not (aOR = 1.22, 95% CI 0.71–2.08, *p* = 0.458).

### Mediation analysis linking ADHD symptoms to subsequent IGD symptoms

**FIGURE 2:**
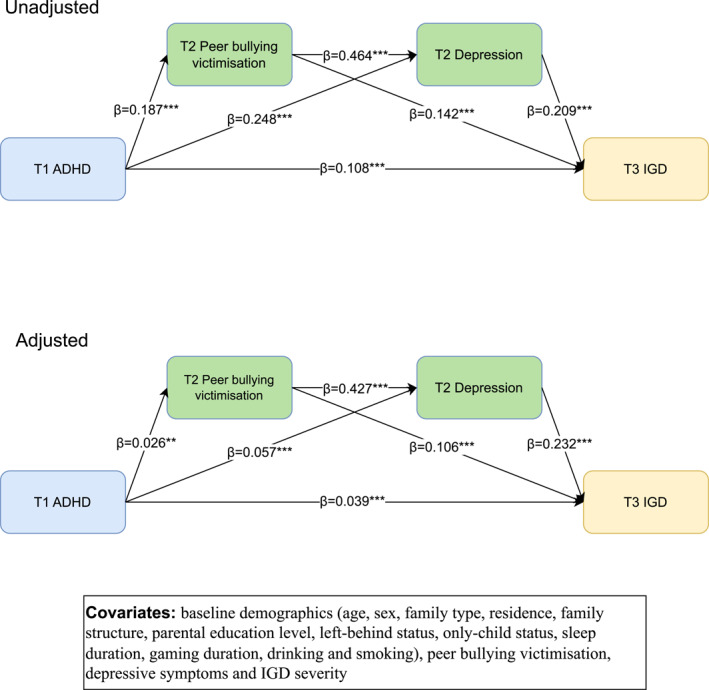
The mediation effect of peer bullying and depression between ADHD symptoms and IGD among the whole sample (*n* = 20 137). ADHD, attention‐deficit/hyperactivity disorder; IGD, internet gaming disorder. **p < 0.01, ***p < 0.001.

Longitudinal path analysis (figure 2) tested a serial mediation model with T1 total ADHD symptoms predicting T3 IGD severity through T2 peer bullying victimisation and T2 depressive symptoms. In the unadjusted model, T1 ADHD symptoms significantly predicted T2 peer bullying victimisation (*β* = 0.187, 95% CI 0.171–0.203, *p* < 0.001) and T2 depressive symptoms (*β* = 0.248, 95% CI 0.234–0.262, *p* < 0.001). T2 peer bullying victimisation significantly predicted T2 depressive symptoms (*β* = 0.464, 95% CI 0.448–0.479, *p* < 0.001). T2 peer bullying victimisation and T2 depressive symptoms both significantly predicted T3 IGD severity (peer bullying: *β* = 0.142, 95% CI 0.124–0.157; depressive symptoms: *β* = 0.209, 95% CI 0.196–0.225, all *p* < 0.001). A significant direct effect of T1 ADHD symptoms on T3 IGD was also observed (*β* = 0.108, 95% CI 0.091–0.126, *p* < 0.001).

Significant indirect pathways included mediation through peer bullying victimisation alone (*β* = 0.026, 95% CI 0.022–0.031), depressive symptoms alone (*β* = 0.052, 95% CI 0.045–0.059) and the serial pathway through bullying then depression (*β* = 0.018, 95% CI 0.015–0.021). The total effect of T1 ADHD symptoms on T3 IGD was significant (*β* = 0.202, 95% CI 0.189–0.221, *p* < 0.001), with total indirect effects accounting for 47.52% of this relationship. After adjusting for baseline covariates and baseline symptoms, all specific indirect pathways remained statistically significant, and the total mediated proportion slightly decreased to 32.76%.

### Difference in the ADHD‐bullying‐depression‐IGD pathway across sex and developmental stages

Multi‐group analysis revealed significant sex differences in the overall mediation model (Δ*χ* ^2^ = 133.257, Δdf = 6, *p* < 0.001). Specifically, the path from baseline total ADHD symptoms to T2 peer bullying victimisation was significant for boys (*β* = 0.034, 95% CI 0.008–0.061, *p* = 0.012) but not for girls (*β* = 0.019, 95% CI –0.007 to 0.044, *p* = 0.149). Consequently, indirect effects mediated solely through peer bullying victimisation (*β* = 0.004, 95% CI 0.001–0.007) and serially through peer bullying followed by depressive symptoms (*β* = 0.003, 95% CI 0.001–0.006) were significant only among boys (table 3). Direct effects of ADHD symptoms on IGD severity were significant in both sexes but stronger in boys (*β* = 0.046, 95% CI 0.020–0.073, *p* = 0.001) than girls (*β* = 0.030, 95% CI 0.003–0.056, *p* = 0.026). Indirect effects mediated solely through depressive symptoms were significant and comparable in magnitude for both boys (*β* = 0.015, 95% CI 0.009–0.021) and girls (*β* = 0.013, 95% CI 0.007–0.018). The total effect of ADHD symptoms on IGD severity was greater in boys (*β* = 0.069, 95% CI 0.042–0.096, *p* < 0.001) than in girls (*β* = 0.046, 95% CI 0.019–0.072, *p* = 0.001).

**TABLE 3:** Subgroup difference across sex and developmental stages

	Sex difference				Developmental difference			
	Boys		Girls		Early (7th grade)		Late (10th grade)	
	*β* (95% CI)	Mediation ratio	*β* (95% CI)	Mediation ratio	*β* (95% CI)	Mediation ratio	*β* (95% CI)	Mediation ratio
Direct effect (ADHD symptoms → IGD severity)	0.046 (0.020–0.073)	‐	0.030 (0.003–0.056)	‐	0.047 (0.023–0.064)		0.027 (−0.002 to 0.055)	
Indirect effect								
ADHD symptoms → peer bullying victimisation → IGD severity	0.004 (0.001–0.007)	5.80%	0.002 (−0.001 to 0.004)	‐	0.003 (0.001–0.005)	4.62%	0.003 (−0.001 to 0.006)	‐
ADHD symptoms → depressive symptoms → IGD severity	0.015 (0.009–0.021)	21.74%	0.013 (0.007–0.018)	28.26%	0.012 (0.006–0.018)	18.46%	0.014 (0.007–0.021)	30.43%
ADHD symptoms → peer bullying victimisation → depressive symptoms → IGD severity	0.003 (0.001–0.006)	4.35%	0.002 (−0.001 to 0.005)	‐	0.003 (0.001–0.005)	4.62%	0.002 (−0.001 to 0.004)	‐
Total indirect effects	0.023 (0.014–0.031)	33.33%	0.016 (0.009–0.024)	‐	0.018 (0.010–0.026)	27.69%	0.018 (0.010–0.026)	‐
Total effects	0.069 (0.042–0.096)	‐	0.046 (0.019–0.072)	‐	0.065 (0.041–0.083)	‐	0.046 (0.015–0.077)	‐

*Note*: Adjusted for baseline demographics (age, sex, family type, residence, family structure, parental education level, left‐behind status, only‐child status, sleep duration, gaming duration, drinking and smoking), peer bullying victimisation, depressive symptoms and IGD severity.

Abbreviations: ADHD, attention‐deficit/hyperactivity disorder; CI, confidence interval; IGD, internet gaming disorder.

Multi‐group analyses by developmental stage revealed significant age‐related heterogeneity. A model constraining all structural paths to be equal across early adolescents (Grade 7) and late adolescents (Grade 10) demonstrated a significantly poorer fit than the unconstrained model (Δ*χ* ^2^ = 43.906, Δdf = 6, *p* < 0.001), indicating meaningful between‐group differences (table 3). Specifically, the path from baseline ADHD symptoms to T2 peer bullying victimisation was significant among early adolescents (*β* = 0.028, *p* = 0.019) but not among late adolescents (*β* = 0.026, *p* = 0.093). Consequently, indirect effects through peer bullying alone (*β* = 0.003, 95% CI 0.001–0.005) and serially through peer bullying followed by depressive symptoms (*β* = 0.003, 95% CI 0.001–0.005) were statistically significant only in early adolescence. The indirect effect mediated solely through depressive symptoms was larger in magnitude (*β* = 0.012, 95% CI 0.006–0.018), accounting for approximately 18.5% of the total effect. The direct effect of ADHD symptoms on IGD severity also remained significant in early adolescence (*β* = 0.047, 95% CI 0.023–0.064, *p* < 0.001).

In contrast, among late adolescents, ADHD symptoms were not significantly associated with subsequent peer bullying victimisation, and neither the indirect effect through peer bullying alone (*β* = 0.003, 95% CI −0.001 to 0.006) nor the serial indirect effect involving both peer bullying and depressive symptoms (*β* = 0.002, 95% CI −0.001 to 0.004) reached statistical significance. However, the indirect pathway mediated through depressive symptoms remained significant (*β* = 0.014, 95% CI 0.007–0.021). The direct effect of ADHD symptoms on IGD severity was attenuated and no longer statistically significant in late adolescence (*β* = 0.027, 95% CI −0.002 to 0.055, *p* = 0.071).

### Sensitivity analysis

#### Alternative mediation structures

In the reversed serial mediation model (ADHD → depressive symptoms → peer bullying → IGD), the total indirect effect remained comparable in magnitude to that observed in the primary model. The indirect pathway mediated solely through depressive symptoms remained statistically significant (*β* = 0.016, 95% CI 0.012–0.022), whereas the indirect effect mediated solely through peer bullying was no longer supported (*β* = −0.001, 95% CI −0.003 to 0.002). The serial indirect effect involving both depressive symptoms and peer bullying remained statistically significant but small in magnitude (*β* = 0.003, 95% CI 0.002–0.005). This pattern indicates that while depressive symptoms consistently function as a proximal mediator of IGD risk, peer bullying is less likely to operate as a downstream mediator following depressive symptoms.

In the parallel mediation model, both peer bullying victimisation (*β* = 0.003, 95% CI 0.001–0.005) and depressive symptoms (*β* = 0.016, 95% CI 0.012–0.020) independently mediated the association between ADHD symptoms and IGD severity. The total indirect effect was similar in magnitude to that observed in the serial mediation models. However, this specification did not account for the interrelationship between peer bullying victimisation and depressive symptoms.

#### Cross‐lagged panel model

To account for autoregressive stability and explicitly evaluate potential reciprocal associations among the study variables, a CLPM was estimated across three waves. The model demonstrated acceptable fit to the data (CFI = 0.987, RMSEA = 0.055, SRMR = 0.015). All constructs showed substantial temporal stability, with significant autoregressive paths across adjacent waves (all *p* < 0.001).

After accounting for autoregressive effects and reciprocal associations, ADHD symptoms remained a consistent prospective predictor of subsequent IGD severity across waves. Specifically, ADHD symptoms at T1 significantly predicted peer bullying victimisation (*β* = 0.028, *p* = 0.003), depressive symptoms (*β* = 0.072, *p* < 0.001) and IGD severity (*β* = 0.051, *p* < 0.001) at T2. In turn, peer bullying victimisation (*β* = 0.036, *p* < 0.001) and depressive symptoms (*β* = 0.060, *p* < 0.001) at T2 independently predicted IGD severity at T3, above prior IGD levels. Correspondingly, the total indirect association between ADHD symptoms at T1 and IGD severity at T3 was statistically significant (*β* = 0.005, 95% CI 0.003–0.007), comprising both simple indirect pathways through peer bullying victimisation and depressive symptoms.

Within the constraints of the three‐wave design, additional process‐level evidence supported the proposed social–emotional ordering. ADHD symptoms at T1 predicted peer bullying victimisation at T2, which subsequently predicted depressive symptoms at T3. The indirect effect was significant (*β* = 0.003, 95% CI 0.001–0.005). Moreover, peer bullying victimisation at T1 predicted depressive symptoms at T2, which in turn predicted IGD severity at T3. The indirect effect was also significant (*β* = 0.005, 95% CI 0.003–0.008). These findings indicate that the component pathways underlying the hypothesised ADHD → bullying → depression → IGD sequence were observable in temporally ordered form, even though a fully time‐separated serial mediation could not be formally tested within a three‐wave CLPM.

In the reverse direction, IGD severity at T1 predicted depressive symptoms, but not peer bullying victimisation, at T2. Although depressive symptoms at T2 subsequently predicted ADHD symptoms at T3, reverse indirect effects involving peer bullying victimisation were not supported. Overall, although bidirectional associations were evident, the forward pathway from ADHD symptoms to later IGD severity was characterised by greater internal coherence and alignment with a theoretically grounded social–emotional process than the reverse direction.

Taken together, these CLPM findings suggest that the association between ADHD symptoms and subsequent IGD severity is not solely attributable to the autoregressive persistence of ADHD symptoms. Rather, even under conservative modelling conditions that explicitly account for temporal stability and reciprocal influences, ADHD symptoms exert a prospective influence on IGD through interpersonal and emotional mechanisms.

#### Parallel‐process latent growth curve model

The parallel‐process latent growth curve model showed acceptable fit to the data (CFI = 0.942, RMSEA = 0.078, SRMR = 0.037). Across the three waves, all four constructs exhibited significant linear declines, with mean slope estimates indicating decreasing trajectories in ADHD symptoms (slope = −0.180, SE = 0.009, *p* < 0.001), peer bullying victimisation (slope = −1.293, SE = 0.030, *p* < 0.001), depressive symptoms (slope = −0.479, SE = 0.020, *p* < 0.001) and IGD severity (slope = −0.616, SE = 0.026, *p* < 0.001).

At the intercept level, ADHD symptoms were strongly correlated with peer bullying victimisation, depressive symptoms and IGD severity, indicating substantial baseline co‐occurrence among neurodevelopmental, interpersonal, emotional and behavioural difficulties. Importantly, significant covariances were also observed among the slopes of all constructs. Adolescents with a slower decline in ADHD symptoms tended to show parallel declines in peer bullying victimisation (slope covariance = 0.598, *p* < 0.001), depressive symptoms (0.868, *p* < 0.001) and IGD severity (0.721, *p* < 0.001), with strong positive correlations also observed among the slopes of these outcomes. These findings indicate that ADHD symptoms, peer bullying victimisation, depressive symptoms and IGD severity share both baseline co‐occurrence and tightly coupled developmental trajectories across adolescence.

## DISCUSSION

To our knowledge, this study represents the largest longitudinal investigation to date examining the prospective association between ADHD symptoms and IGD among Chinese adolescents. It is also the first to test the sequential mediating roles of peer bullying victimisation and depressive symptoms within a three‐wave longitudinal framework. Our findings demonstrate both direct and indirect effects of ADHD symptoms on subsequent IGD severity, with peer bullying victimisation and depressive symptoms jointly accounting for approximately one‐third of the total effect. Notably, meaningful heterogeneity emerged across sex and developmental stage. Peer bullying served as a mediator of the ADHD–IGD association only among boys and early adolescents, whereas depressive symptoms consistently mediated this association across sexes and age groups. These results suggest that social victimisation may represent a developmentally and socially specific pathway linking ADHD symptoms to problematic gaming, whereas affective dysregulation constitutes a more pervasive mechanism throughout adolescence. A series of sensitivity analyses further supported the robustness and specificity of the proposed model.

### Main findings

Our results corroborate and extend the growing body of literature on the strong and independent association between ADHD symptoms and IGD.^6^ Several mechanisms may explain the observed direct effect of ADHD symptoms on IGD. First, ADHD symptoms are associated with high sensation seeking, characterised by a propensity to seek novel, varied, complex and intense experiences and a willingness to take risks.^46^ This trait may predispose individuals to seek the immediate and variable reinforcement inherent in video games, which can be more rewarding than less stimulating real‐world activities. Secondly, lower frustration tolerance and emotional dysregulation, common in ADHD,^47^ could lead adolescents to prefer the controllable challenges and clear reward structures of games over potentially frustrating real‐world social or academic tasks. Furthermore, executive function deficits in ADHD,^48^ including impaired self‐control and planning, can directly hinder an adolescent's ability to regulate gaming time and resist excessive play. Finally, from a biological perspective, deficits in the reward system in ADHD (e.g., impaired functional connectivity in reward circuits) could also serve as a vulnerability factor for IGD.^49^

The findings also provide novel insights into the psychological mechanisms underlying the link between ADHD symptoms and IGD. Consistent with the dual failure and compensatory internet use models, adolescents with elevated ADHD symptoms appear more vulnerable to experiencing peer bullying, which in turn exacerbates depressive symptoms. These internalising difficulties may then increase reliance on gaming as a maladaptive coping strategy to escape negative affect or seek compensatory rewards and social connection, ultimately contributing to increased IGD severity. These findings align with mediation pathways observed between ADHD symptoms and other negative outcomes, such as suicidality and psychotic experiences.^50^, ^51^ Collectively, these results underscore that peer bullying victimisation and depressive symptoms represent critical intervention targets for mitigating not only IGD risk but also potentially other forms of mental distress among adolescents with ADHD symptoms.

The observed serial pathway from ADHD symptoms to peer bullying victimisation, depressive symptoms and subsequent IGD may also be interpreted within a neurophysiological framework. ADHD is characterised by deficits in executive control and heightened reward sensitivity,^52^ which have been linked to altered fronto‐striatal functioning and reduced regulatory capacity in socially demanding contexts. These neurocognitive features may increase susceptibility to peer difficulties and victimisation. Peer bullying, in turn, represents a potent chronic social stressor that can amplify limbic reactivity and disrupt affective regulation systems,^53^, ^54^ thereby increasing vulnerability to depressive symptoms. Excessive gaming may subsequently emerge as a compensatory strategy to regulate negative affect and to engage hyper‐responsive reward systems, consistent with neurobiological models of behavioural addiction.^55^, ^56^ Although neural processes were not directly measured in the present study, this interpretation provides a biologically plausible substrate underlying the observed psychosocial cascade.

It is also important to consider how bullying victimisation may impair self‐esteem and self‐representation, thereby fostering self‐focused rumination—a core feature of depression. Neuroimaging research has shown that maladaptive self‐referential processing and rumination are associated with dysfunctional activity within the default mode network,^57^ which plays a central role in self‐evaluation and internally directed thought. Recent evidence further suggests that alterations in the default mode network activity may mediate the effects of biological stress‐related markers on rumination and depressive symptoms.^58^ From this perspective, bullying may contribute to depression not only through overt emotional distress but also by disrupting neural systems supporting adaptive self‐processing. Although the present three‐wave design does not allow for a fully time‐separated test of this neural sequence, acknowledging this possibility helps situate the bullying–depression link within a broader biopsychosocial framework.

Our findings also revealed a significant sex difference in the mediation model, particularly regarding the role of peer bullying victimisation. The indirect effects mediated through peer bullying alone and serially through bullying followed by depressive symptoms were significant only among boys. These results contrast with a previous European study, which found the pathway ADHD symptoms → peer bullying victimisation → depressive symptoms was significant only in girls.^59^ Several other studies, conversely, have reported no sex difference in the relationship between ADHD symptoms and bullying victimisation.^60^

Sex‐specific patterns in peer stress responses and emotional processing may help explain why peer bullying acts as a stronger mediator among boys in our study. Large epidemiological studies indicate differential profiles of bullying involvement in adolescents with ADHD by sex,^61^ whereas meta‐analytic evidence shows that peer victimisation tends to be more strongly associated with depressive outcomes in females.^62^ For boys, peer victimisation may represent a direct threat to self‐worth and social competence, which is particularly difficult to regulate under traditional masculine norms emphasising competence and emotional restraint. In this context, immersive gaming environments may serve as a compensatory arena in which boys can restore a sense of mastery, control and social recognition through in‐game achievements and avatar identification.^63^ Consistent with this interpretation, prior studies have shown that boys are more prone to overvalue the reward‐related and self‐enhancing aspects of gaming, including beliefs that gaming increases personal control and garners peer respect.^64^ Together, these findings provide a plausible explanation for the observed sex‐specific mediation pattern, in which peer bullying played a more prominent role among boys, whereas depressive symptoms operated as a more general mediator across sexes. Future studies integrating neurobiological measures are warranted to further elucidate the shared neural mechanisms underlying these sex‐differentiated social–emotional pathways.

### Limitations

This study has several limitations. First, although the three‐wave longitudinal design represents a clear methodological advance over the predominantly cross‐sectional or two‐wave studies in this field, it nonetheless imposes constraints on the temporal separation of predictors, mediators and outcomes. Specifically, peer bullying victimisation and depressive symptoms were assessed at the same wave, which necessitated theoretically informed assumptions regarding their within‐wave ordering in the serial mediation model. Although this ordering was grounded in established developmental frameworks and further supported by multiple sensitivity analyses, a four‐wave (or more) design would allow each component of the hypothesised cascade to be temporally isolated, thereby providing a more stringent test of sequential mediation and reducing reliance on within‐wave assumptions. Moreover, the observational nature of the data precludes drawing definitive causal conclusions. Second, the study's reliance on self‐report measures constitutes a significant limitation, particularly given that ADHD and IGD are characterised by externalising behaviours. In clinical practice, third‐party evaluations (e.g., from parents or teachers) are often preferred to ensure objective assessment, as adolescents may lack the necessary insight to accurately report the severity of their behavioural symptoms. Furthermore, our assessment of ADHD utilised a brief screening tool (the SDQ) rather than a formal diagnostic interview. Although valid for large‐scale epidemiological screening, this measure captures symptom severity rather than a confirmed clinical diagnosis and may not reflect the full complexity of the disorder. Consequently, our findings should be interpreted as associations with symptom dimensions rather than clinically diagnosed conditions. MPVS focused solely on traditional peer bullying victimisation and did not assess cyberbullying, an increasingly relevant form of peer aggression among adolescents that may also mediate the pathway to IGD. Third, the study was conducted within a specific developmental period (early to mid‐adolescence) and in a single city in China, which may limit the generalisability of findings to different age groups or diverse cultural contexts. Finally, despite controlling for baseline symptoms and a comprehensive set of covariates, the possibility of unmeasured confounding factors (e.g., genetic predispositions, school environment, parenting styles) influencing the observed associations remains. Future research should address these limitations by incorporating multi‐informant data, standardised clinical interviews, recruiting broader and more diverse populations and utilising more comprehensive measures where feasible to further validate and extend these findings.

### Implications

Our study offers significant academic and clinical implications. Academically, our findings advance the understanding of this comorbidity by demonstrating that the relationship is not solely direct but is significantly mediated by interpersonal (peer bullying victimisation) and emotional (depressive symptoms) factors, providing empirical support for the dual failure and compensatory internet use models. Clinically, these results underscore the necessity of comprehensive assessments for adolescents with ADHD, extending beyond core ADHD symptoms to include evaluation for peer bullying victimisation and depressive symptoms, which represent critical points for intervention. The observed sex‐ and age‐related differences further indicate that uniform intervention strategies may be suboptimal. In particular, the salience of peer bullying among boys and early adolescents suggests that preventive efforts for these groups should prioritise social skills training, peer relationship interventions and school‐based anti‐bullying programmes, whereas emotion‐focused interventions may be broadly relevant across adolescence.

## CONCLUSION

ADHD symptoms are prospectively associated with IGD severity in adolescents through both direct and indirect pathways. The indirect association operates sequentially through increased peer bullying victimisation and depressive symptoms, with this pathway being particularly evident among boys and early adolescents. These findings underscore the need for developmentally and sex‐sensitive prevention and intervention strategies that address both social and emotional vulnerabilities in adolescents with ADHD.

## AUTHOR CONTRIBUTIONS

Yanhui Liao contributed to all aspects of the study. Pu Peng contributed to the analysis and interpretation of data, statistical analysis, and the drafting of the manuscript. Zhangming Chen, Jinsong Tang, and Xiaogang Chen contributed to the study design. Ying He, Jinguang Li, Aijun Liao, Linlin Zhao, Silan Ren, Shanshan Chen, Xu Shao, Ruini He, Yudiao Liang, and Youguo Tan contributed to the data acquisition. All authors have reviewed, revised and approved the final manuscript.

## CONFLICT OF INTEREST STATEMENT

The authors declare no conflicts of interest.

## ETHICS STATEMENT

This study was performed in line with the principles of the Declaration of Helsinki. The study was approved by the Ethics Committee of the Zigong Mental Health Center (Approval No. 2020‐8‐01).

## CONSENT TO PARTICIPATE

Informed consent was obtained from all participants and their parents for those under 18 years old.

## Supporting information

Tables S1–S5

## Data Availability

The data was available on request from the corresponding author.

## References

[1] Coutelle R , Balzer J , Rolling J , Lalanne L . Problematic gaming, psychiatric comorbidities, and adolescence: a systematic review of the literature. Addict Behav. 2024;157:108091. 10.1016/j.addbeh.2024.108091 38901145

[2] Düll L , Müller A , Steins‐Loeber S . Negative consequences experienced by individuals with gaming disorder symptoms: a systematic review of available longitudinal studies. Curr Addict Rep. 2024;11:528–550. 10.1007/s40429-024-00554-2

[3] Sonuga‐Barke EJS , Becker SP , Bölte S , Castellanos FX , Franke B , Newcorn JH , et al. Annual Research Review: perspectives on progress in ADHD science – from characterization to cause. J Child Psychol Psychiatry. 2023;64:506–532. 10.1111/jcpp.13696 36220605 PMC10023337

[4] Garg S , Kharb A , Verma D , Antil R , Khanna B , Sihag R , et al. The mediating role of sleep quality on the relationship between internet gaming disorder and perceived stress and suicidal behaviour among Indian medical students. Gen Psychiatr. 2023;36(3):e100997. 10.1136/gpsych-2022-100997 37304212 PMC10254597

[5] Koncz P , Demetrovics Z , Takacs ZK , Griffiths MD , Nagy T , Király O . The emerging evidence on the association between symptoms of ADHD and gaming disorder: a systematic review and meta‐analysis. Clin Psychol Rev. 2023;106:102343. 10.1016/j.cpr.2023.102343 37883910

[6] Thorell LB , Burén J , Ström Wiman J , Sandberg D , Nutley SB . Longitudinal associations between digital media use and ADHD symptoms in children and adolescents: a systematic literature review. Eur Child Adolesc Psychiatry. 2024;33(8):2503–2526. 10.1007/s00787-022-02130-3 36562860 PMC11272698

[7] Hygen BW , Skalická V , Stenseng F , Belsky J , Steinsbekk S , Wichstrøm L . The co‐occurrence between symptoms of internet gaming disorder and psychiatric disorders in childhood and adolescence: prospective relations or common causes? J Child Psychol Psychiatry. 2020;61:890–898. 10.1111/jcpp.13289 32623728

[8] Wichstrøm L , Stenseng F , Belsky J , von Soest T , Hygen BW . Symptoms of internet gaming disorder in youth: predictors and comorbidity. J Abnorm Child Psychol. 2019;47(1):71–83. 10.1007/s10802-018-0422-x 29623484 PMC6329732

[9] Hong YN , Hwang H , Starcevic V , Choi TY , Kim TH , Han DH . Which is more stable and specific: DSM‐5 internet gaming disorder or ICD‐11 gaming disorder? A longitudinal study. Psychiatry Clin Neurosci. 2023;77(4):213–222. 10.1111/pcn.13522 36562926

[10] Lee J , Bae S , Kim BN , Han DH . Impact of attention‐deficit/hyperactivity disorder comorbidity on longitudinal course in internet gaming disorder: a 3‐year clinical cohort study. J Child Psychol Psychiatry. 2021;62:1110–1119. 10.1111/jcpp.13380 33751554

[11] Park JH , Lee YS , Sohn JH , Han DH . Effectiveness of atomoxetine and methylphenidate for problematic online gaming in adolescents with attention deficit hyperactivity disorder. Hum Psychopharmacol. 2016;31(6):427–432. 10.1002/hup.2559 27859666

[12] de Sá RRC , Coelho S , Parmar PK , Johnstone S , Kim HS , Tavares H . A systematic review of pharmacological treatments for internet gaming disorder. Psychiatry Investig. 2023;20(8):696–706. 10.30773/pi.2022.0297 PMC10460977 37559452

[13] Jeong H , Yim HW , Lee S‐Y , Lee HK , Potenza MN , Jo S‐J , et al. Low self‐control and aggression exert serial mediation between inattention/hyperactivity problems and severity of internet gaming disorder features longitudinally among adolescents. J Behav Addict. 2020;9(2):401–409. 10.1556/2006.2020.00039 32634112 PMC8939404

[14] Boutin S , Roy V , St‐Pierre RA , Déry M , Lemelin JP , Martin‐Storey A , et al. The longitudinal association between externalizing and internalizing problems: an exploration of the dual failure model. Dev Psychol. 2020;56(7):1372–1384. 10.1037/dev0000935 32352825

[15] Gong X , Zhou J , Huebner ES , Tian L . Longitudinal association and mediating mechanism between externalizing and internalizing problems among children: a within‐person analysis. J Clin Child Adolesc Psychol. 2024;53(4):637–651. 10.1080/15374416.2022.2158836 36625685

[16] Kardefelt‐Winther D . A conceptual and methodological critique of internet addiction research: towards a model of compensatory internet use. Comput Hum Behav. 2014;31:351–354. 10.1016/j.chb.2013.10.059

[17] Mizuno Y , Yamashita M , Shou Q , Hamatani S , Cai W . A brief review of MRI studies in patients with attention‐deficit/hyperactivity disorder and future perspectives. Brain Dev. 2025;47(2):104340. 10.1016/j.braindev.2025.104340 40043540

[18] Zheng Y‐B , Zhang S‐N , Tang H‐D , Wang S‐W , Lin X , Bao Y‐P , et al. Gaming disorder: neural mechanisms and ongoing debates. J Behav Addict. 2025;14(1):55–78. 10.1556/2006.2024.00071 39786382 PMC11974403

[19] Bertollo AG , Puntel CF , da Silva BV , Martins M , Bagatini MD , Ignácio ZM . Neurobiological relationships between neurodevelopmental disorders and mood disorders. Brain Sci. 2025;15(3):307. 10.3390/brainsci15030307 40149827 PMC11940368

[20] Grimm O , van Rooij D , Hoogman M , Klein M , Buitelaar J , Franke B , et al. Transdiagnostic neuroimaging of reward system phenotypes in ADHD and comorbid disorders. Neurosci Biobehav Rev. 2021;128:165–181. 10.1016/j.neubiorev.2021.06.025 34144113

[21] Soler‐Gutiérrez A‐M , Pérez‐González J‐C , Mayas J . Evidence of emotion dysregulation as a core symptom of adult ADHD: a systematic review. PLoS One. 2023;18:e0280131. 10.1371/journal.pone.0280131 36608036 PMC9821724

[22] Chen C , Dai S , Shi L , Shen Y , Ou J . Associations between attention deficit/hyperactivity disorder and internet gaming disorder symptoms mediated by depressive symptoms and hopelessness among college students. Neuropsychiatr Dis Treat. 2021;17:2775–2782. 10.2147/NDT.S325323 34465993 PMC8403024

[23] Peng P , Chen Z , Ren S , He Y , Li J , Liao A , et al. Sex difference in the longitudinal association between depressive symptoms and internet gaming disorder among Chinese adolescents: an explanatory analysis at the aggregate and symptom level. Addict Behav. 2025;172:108499. 10.1016/j.addbeh.2025.108499 40974915

[24] Chen Z , Ren S , He R , Liang Y , Tan Y , Liu Y , et al. Prevalence and associated factors of depressive and anxiety symptoms among Chinese secondary school students. BMC Psychiatry. 2023;23(1):580. 10.1186/s12888-023-05068-1 37563573 PMC10413612

[25] Peng P , Chen Z , Ren S , Liu Y , He R , Liang Y , et al. Determination of the cutoff point for Smartphone Application‐Based Addiction Scale for adolescents: a latent profile analysis. BMC Psychiatry. 2023;23(1):675. 10.1186/s12888-023-05170-4 37716941 PMC10504767

[26] Peng P , Chen Z , Ren S , Liu Y , Li J , Liao A , et al. Association between school bullying victimization and e‐cigarette use and its sex difference: evidence from Chinese adolescents. J Smok Cessat. 2025;20(1):0. 10.48130/jsc-0025-0001

[27] Peng P , Jin J , Chen Z , Ren S , He Y , Li J , et al. Impaired sleep quality mediates the relationship between internet gaming disorder and conduct problems among adolescents: a three‐wave longitudinal study. Child Adolesc Psychiatry Ment Health. 2025;19(1):26. 10.1186/s13034-025-00889-2 40119352 PMC11929296

[28] Peng P , Chen Z , Ren S , He Y , Li J , Liao A , et al. Trajectory of internet gaming disorder among Chinese adolescents: course, predictors, and long‐term mental health outcomes. J Behav Addict. 2025;14(2):846–860. 10.1556/2006.2025.00054 40549959 PMC12231443

[29] Peng P , Chen Z , Ren S , He Y , Li J , Liao A , et al. The effect of bullying victimization trajectory on internet gaming disorder and the mediating role of impaired resilience: a three‐wave cohort study among Chinese adolescents. BMC Psychiatry. 2025;25(1):1180. 10.1186/s12888-025-07641-2 41275164 PMC12750727

[30] Goodman R . The Strengths and Difficulties Questionnaire: a research note. J Child Psychol Psychiatry. 1997;38(5):581–586. 10.1111/j.1469-7610.1997.tb01545.x 9255702

[31] Liu S‐K , Chien Y‐L , Shang C‐Y , Lin CH , Liu YC , Gau SSF . Psychometric properties of the Chinese version of Strength and Difficulties Questionnaire. Compr Psychiatry. 2013;54(6):720–730. 10.1016/j.comppsych.2013.01.002 23433222

[32] Wang J‐L , Yin X‐Q , Wang H‐Z , King DL , Rost DH . The longitudinal associations between internet addiction and ADHD symptoms among adolescents. J Behav Addict. 2024;13(1):191–204. 10.1556/2006.2023.00080 38206342 PMC10988408

[33] Peng P , Chen Z , Ren S , He Y , Li J , Liao A , et al. Internet gaming disorder predicts the incidence, persistence, and worsening of suicidal ideation: a population‐based cohort study of 96,158 Chinese adolescents. J Affect Disord. 2025;379:186–193. 10.1016/j.jad.2025.03.029 40081581

[34] Mynard H , Joseph S . Development of the multidimensional peer‐victimization scale. Aggress Behav. 2000;26(2):169–178. 10.1002/(SICI)1098-2337(2000)26:2<169::AID-AB3>3.0.CO;2-A

[35] Li X , Ng TK , Lee TH , Li CN . Peer victimization among Chinese adolescents: a longitudinal validation study. Psychol Assess. 2024;36(1):53–65. 10.1037/pas0001289 37917496

[36] Levis B , Benedetti A , Thombs BD , DEPRESsion Screening Data (DEPRESSD) Collaboration . Accuracy of Patient Health Questionnaire‐9 (PHQ‐9) for screening to detect major depression: individual participant data meta‐analysis. BMJ. 2019;365:l1476. 10.1136/bmj.l1476 30967483 PMC6454318

[37] Zhang Y‐L , Liang W , Chen Z.‐M , Zhang HM , Zhang JH , Weng XQ , et al. Validity and reliability of Patient Health Questionnaire‐9 and Patient Health Questionnaire‐2 to screen for depression among college students in China. Asia Pac Psychiatry. 2013;5(4):268–275. 10.1111/appy.12103 24123859

[38] Xu S , Ju Y , Wei X , Ou W , Ma M , Lv G , et al. Network analysis of suicide ideation and depression‐anxiety symptoms among Chinese adolescents. Gen Psychiatr. 2024;37(2):e101225. 10.1136/gpsych-2023-101225 38562407 PMC10982688

[39] Jiang W , Yao J , Wang Y , Su S , Zheng Z , Yang Y , et al. Efficacy of dynamic interpersonal therapy in improving mentalising in patients with major depressive disorder and the mediating effect of mentalising on changes in depressive symptoms. Gen Psychiatr. 2025;38(1):e101774. 10.1136/gpsych-2024-101774 40017493 PMC11865740

[40] Chen I‐H , Strong C , Lin Y‐C , Tsai M‐C , Leung H , Lin C‐Y , et al. Time invariance of three ultra‐brief internet‐related instruments: Smartphone Application‐Based Addiction Scale (SABAS), Bergen Social Media Addiction Scale (BSMAS), and the nine‐item internet Gaming Disorder Scale‐Short Form (IGDS‐SF9) (Study Part B). Addict Behav. 2020;101:105960. 10.1016/j.addbeh.2019.04.018 31072648

[41] Pontes HM , Griffiths MD . Measuring DSM‐5 internet gaming disorder: development and validation of a short psychometric scale. Comput Hum Behav. 2015;45:137–143. 10.1016/j.chb.2014.12.006

[42] Hou J , Xiao Q , Zhou M , Xiao L , Yuan M , Zhong N , et al. Lower synaptic density associated with gaming disorder: an 18F‐SynVesT‐1 PET imaging study. Gen Psychiatr. 2023;36(5):e101112. 10.1136/gpsych-2023-101112 37829163 PMC10565144

[43] Qin L , Cheng L , Hu M , Liu Q , Tong J , Hao W , et al. Clarification of the cut‐off score for nine‐item Internet Gaming Disorder Scale‐Short Form (IGDS9‐SF) in a Chinese context. Front Psychiatry. 2020;11:470. 10.3389/fpsyt.2020.00470 32528331 PMC7262730

[44] Sun M , Scherffius A , Sun M , Chen C , Wang D . Insomnia symptoms as a mediator between school connectedness and suicidal ideation in Chinese adolescents: a three‐wave longitudinal model. Early Interv Psychiatry. 2025;19(1):e13579. 10.1111/eip.13579 38783351

[45] Wu R , Niu Q , Wang Y , Dawa Y , Guang Z , Song D , et al. The impact of problematic smartphone use on sleep quality among Chinese young adults: investigating anxiety and depression as mediators in a three‐wave longitudinal study. Psychol Res Behav Manag. 2024;17:1775–1786. 10.2147/PRBM.S455955 38707963 PMC11067928

[46] Dalbudak E , Evren C , Aldemir S , Taymur I , Evren B , Topcu M . The impact of sensation seeking on the relationship between attention deficit/hyperactivity symptoms and severity of Internet addiction risk. Psychiatry Res. 2015;228(1):156–161. 10.1016/j.psychres.2015.04.035 25962354

[47] Seymour KE , Macatee R , Chronis‐Tuscano A . Frustration tolerance in youth with ADHD. J Atten Disord. 2019;23(11):1229–1239. 10.1177/1087054716653216 27282378 PMC6541529

[48] Wang M , Yu J , Kim H‐D , Cruz AB . Neural correlates of executive function and attention in children with ADHD: an ALE meta‐analysis of task‐based functional connectivity studies. Psychiatry Res. 2025;345:116338. 10.1016/j.psychres.2024.116338 39947841

[49] Gao X , Zhang M , Yang Z , Wen M , Huang H , Zheng R , et al. Structural and functional brain abnormalities in internet gaming disorder and attention‐deficit/hyperactivity disorder: a comparative meta‐analysis. Front Psychiatry. 2021;12:679437. 10.3389/fpsyt.2021.679437 34276447 PMC8281314

[50] Hennig T , Jaya ES , Lincoln TM . Bullying mediates between attention‐deficit/hyperactivity disorder in childhood and psychotic experiences in early adolescence. Schizophr Bull. 2017;43:1036–1044. 10.1093/schbul/sbw139 27803356 PMC5581899

[51] Lin P‐I , Wu WT , Azasu EK , Wong TY . Pathway from attention‐deficit/hyperactivity disorder to suicide/self‐harm. Psychiatry Res. 2024;337:115936. 10.1016/j.psychres.2024.115936 38705042

[52] Arnsten AFT , Rubia K . Neurobiological circuits regulating attention, cognitive control, motivation, and emotion: disruptions in neurodevelopmental psychiatric disorders. J Am Acad Child Adolesc Psychiatry. 2012;51(4):356–367. 10.1016/j.jaac.2012.01.008 22449642

[53] Perino MT , Guassi Moreira JF , Telzer EH . Links between adolescent bullying and neural activation to viewing social exclusion. Cogn Affect Behav Neurosci. 2019;19(6):1467–1478. 10.3758/s13415-019-00739-7 31292887 PMC6864266

[54] Lim L , Rubia K , Lukito S . Common neural correlates of disgust processing in childhood maltreatment and peer victimisation. BJPsych Open. 2024;10(6):e185. 10.1192/bjo.2024.767 39465580 PMC11698183

[55] Brand M , Müller A , Wegmann E , Antons S , Brandtner A , Müller SM , et al. Current interpretations of the I‐PACE model of behavioral addictions. J Behav Addict. 2025;14:1–17. 10.1556/2006.2025.00020 40063161 PMC11974429

[56] Brand M , Wegmann E , Stark R , Müller A , Wölfling K , Robbins TW , et al. The Interaction of Person‐Affect‐Cognition‐Execution (I‐PACE) model for addictive behaviors: update, generalization to addictive behaviors beyond internet‐use disorders, and specification of the process character of addictive behaviors. Neurosci Biobehav Rev. 2019;104:1–10. 10.1016/j.neubiorev.2019.06.032 31247240

[57] Zhou H.‐X , Chen X , Shen Y.‐Q , Li L , Chen NX , Zhu ZC , et al. Rumination and the default mode network: meta‐analysis of brain imaging studies and implications for depression. Neuroimage. 2020;206:116287. 10.1016/j.neuroimage.2019.116287 31655111

[58] Jia F , Chen X , Wang X , Quan C , Ruan J , Huang Y , et al. Activity of the default mode network mediates the effect of peripheral plasma glial cell line‐derived neurotrophic factor levels on rumination in major depressive disorder patients. Psychoradiology. 2025;5:kkaf014. 10.1093/psyrad/kkaf014 40586055 PMC12202882

[59] Roy A , Hartman CA , Veenstra R , Oldehinkel AJ . Peer dislike and victimisation in pathways from ADHD symptoms to depression. Eur Child Adolesc Psychiatry. 2015;24(8):887–895. 10.1007/s00787-014-0633-9 25348085

[60] Stenseng F , Skalická V , Skaug SS , Belsky J , Wichstrøm L . Attention‐deficit hyperactivity disorder symptoms and bullying victimization from childhood to adolescence – a within‐person cross‐lagged approach. Dev Psychopathol. 2024;37(3):1–11. 10.1017/S0954579424001251 39363713

[61] Kim D , Choi YH , Kim J . ADHD and sex differences in school bullying victimization and perpetration based on inverse probability treatment weighting. Child Psychiatry Hum Dev. 2025. Published online first. 10.1007/s10578-025-01930-3 41217568

[62] Song Q , Yuan T , Hu Y , Liu X , Fei J , Zhao X , et al. The effect of peer victimization during adolescence on depression and gender differences: a systematic review and meta‐analysis. Trauma Violence Abuse. 2024;25(4):2862–2876. 10.1177/15248380241227538 38347760

[63] Ji Y , Wah LTK , Dai X , Du N , Keung Wong DF . Gaming disorder: unraveling the role of problematic affective, cognitive, and executive functioning – a systematic review and meta‐analytic structural equation modeling. Comput Hum Behav. 2024;159:108348. 10.1016/j.chb.2024.108348

[64] Yu Y , Mo PKH , Zhang J , Li J , Lau JT . Why is internet gaming disorder more prevalent among Chinese male than female adolescents? The role of cognitive mediators. Addict Behav. 2021;112:106637. 10.1016/j.addbeh.2020.106637 32919322

