# Supplementary material for: Peer bullying victimisation and depressive symptoms as serial mediators between attention‐deficit/hyperactivity disorder symptoms and internet gaming disorder among Chinese adolescents: A three‐wave longitudinal study
Source: Gen Psychiatr. 2026 Mar 23;39(1):e70012. doi: 10.1002/gps3.70012 (PMC13015837; doi:10.1002/gps3.70012)
Supplement: Supplementary file 1 — Tables S1–S5 [file GPS3-39-e70012-s001.docx]

**Table S1. Baseline sample characteristics across three waves**

| Variables | T1 (n = 20 137) | T2 (n = 15 061) | T3 (n = 14 706) | Statistic | *P^1^* |
| --- | --- | --- | --- | --- | --- |
|  |  |  |  |  |  |
| Age, Mean (SD) | 13.40 (1.45) | 13.48 (1.47) | 13.44 (1.46) | F=11.15 | <0.001 |
| Gender, n (%) |  |  |  | χ²=18.09 | <0.001 |
| Boys | 9952 (49.42) | 7170 (47.61) | 7341 (49.92) |  |  |
| Girls | 10 185 (50.58) | 7891 (52.39) | 7365 (50.08) |  |  |
| Residence, n (%) |  |  |  | χ²=36.24 | <0.001 |
| Country | 12 900 (64.06) | 9819 (65.19) | 9877 (67.16) |  |  |
| Urban | 7237 (35.94) | 5242 (34.81) | 4829 (32.84) |  |  |
| Only child, n (%) |  |  |  | χ²=21.69 | <0.001 |
| No | 15 606 (77.50) | 11 872 (78.83) | 11 692 (79.50) |  |  |
| Yes | 4531 (22.50) | 3189 (21.17) | 3014 (20.50) |  |  |
| Left-behind child, n (%) |  |  |  | χ²=5.37 | 0.068 |
| No | 13 383 (66.46) | 9912 (65.81) | 9600 (65.28) |  |  |
| Yes | 6754 (33.54) | 5149 (34.19) | 5106 (34.72) |  |  |
| Father education level, n (%) |  |  |  | χ²=13.00 | 0.002 |
| Below high school | 15 453 (76.74) | 11 677 (77.53) | 11 525 (78.37) |  |  |
| High school or above | 4684 (23.26) | 3384 (22.47) | 3181 (21.63) |  |  |
| Mother education level, n (%) |  |  |  | χ²=20.15 | <0.001 |
| Below high school | 16 032 (79.61) | 12 159 (80.73) | 11 987 (81.51) |  |  |
| High school or above | 4105 (20.39) | 2902 (19.27) | 2719 (18.49) |  |  |
| Family type, n (%) |  |  |  | χ²=1.89 | 0.388 |
| Nuclear family | 15 977 (79.34) | 12 024 (79.84) | 11 744 (79.86) |  |  |
| Single parent or remarried | 4160 (20.66) | 3037 (20.16) | 2962 (20.14) |  |  |
| Alcohol use, n (%) |  |  |  | χ²=2.46 | 0.292 |
| Yes | 3395 (16.86) | 2446 (16.24) | 2426 (16.50) |  |  |
| No | 16 742 (83.14) | 12 615 (83.76) | 12 280 (83.50) |  |  |
| Smoking, n (%) |  |  |  | χ²=0.56 | 0.756 |
| Yes | 1394 (6.92) | 1012 (6.72) | 1004 (6.83) |  |  |
| No | 18 743 (93.08) | 14 049 (93.28) | 13 702 (93.17) |  |  |
| Grade, n (%) |  |  |  | χ²=28.18 | <0.001 |
| 7 | 13 361 (66.35) | 9583 (63.63) | 9571 (65.08) |  |  |
| 10 | 6776 (33.65) | 5478 (36.37) | 5135 (34.92) |  |  |
| Gaming time, mean (SD) | 11.20 (19.31) | 10.91 (18.59) | 11.18 (18.90) | F=1.13 | 0.324 |
| MPVS, mean (SD) | 6.39 (7.58) | 6.22 (7.43) | 6.29 (7.50) | F=2.37 | 0.094 |
| Sleep duration, mean (SD) | 7.12 (1.46) | 7.10 (1.45) | 7.15 (1.44) | F=3.66 | 0.026 |
| Internet gaming disorder, n (%) |  |  |  | χ²=1.59 | 0.451 |
| No | 19 621 (97.44) | 14 705 (97.64) | 14 351 (97.59) |  |  |
| Yes | 516 (2.56) | 356 (2.36) | 355 (2.41) |  |  |
| Hyperactivity/inattention, n (%) |  |  |  | χ²=1.58 | 0.454 |
| No | 18 155 (90.16) | 13 583 (90.19) | 13 314 (90.53) |  |  |
| Yes | 1982 (9.84) | 1478 (9.81) | 1392 (9.47) |  |  |
| Depressive symptoms, n (%) |  |  |  | χ²=4.31 | 0.116 |
| No | 15 820 (78.56) | 11 912 (79.09) | 11 686 (79.46) |  |  |
| Yes | 4317 (21.44) | 3149 (20.91) | 3020 (20.54) |  |  |

^1^ ANOVA tests or chi-square tests

Abbreviations: ADHD, attention-deficit/hyperactivity disorder; IGD, internet gaming disorder; SD, standard deviation.

**Table S2. Reversed serial mediation model (T1ADHD symptom→ T2 depressive symptoms → T2 peer bullying → T3 IGD)**

| **Paths** | **β (95% CI)** |
| --- | --- |
| **Direct effect (ADHD → IGD)** | 0.039 (0.023 to 0.064) |
| **Indirect effect** |  |
| ADHD → Depressive symptoms → IGD | 0.016 (0.012 to 0.022) |
| ADHD → Peer bullying → IGD | −0.001 (−0.003 to 0.002) |
| ADHD → Depressive symptoms → Peer bullying → IGD | 0.003 (0.002 to 0.005) |
| **Total indirect effects** | 0.019 (0.014 to 0.026) |
| **Total effects** | 0.058 (0.041 to 0.083) |

Adjusted for baseline demographics (age, sex, family type, residence, family structure, parental education level, left-behind status, only-child status, sleep duration, gaming duration, drinking and smoking), peer bullying victimisation, depressive symptoms and IGD severity.

Abbreviations: ADHD, attention-deficit/hyperactivity disorder; CI, confidence interval; IGD, internet gaming disorder.

**Table S3. Parallel mediation model (peer bullying and depressive symptoms as independent mediators)**

| **Paths** | **β (95% CI)** |
| --- | --- |
| **Direct effect (ADHD → IGD)** | 0.039 (0.020 to 0.058) |
| **Indirect effect** |  |
| ADHD → Depressive symptoms → IGD | 0.016 (0.011 to 0.020) |
| ADHD → Peer bullying → IGD | 0.003 (0.001 to 0.005) |
| **Total indirect effects** | 0.019 (0.013 to 0.024) |
| **Total effects** | 0.058 (0.038 to 0.077) |

Adjusted for baseline demographics (age, sex, family type, residence, family structure, parental education level, left-behind status, only-child status, sleep duration, gaming duration, drinking and smoking), peer bullying victimisation, depressive symptoms and IGD severity.

Abbreviations: ADHD, attention-deficit/hyperactivity disorder; CI, confidence interval; IGD, internet gaming disorder.

**Table S4. Cross-lagged panel model examining reciprocal associations and indirect pathways across three waves (standardised estimates)**

| **Path** | **Wave 1 → Wave 2** | **Wave 2 → Wave 3** |
| --- | --- | --- |
| **Autoregressive paths** |  |  |
| ADHD → ADHD | 0.314 (0.009)^***^ | 0.430 (0.010)^***^ |
| Bullying → Bullying | 0.359 (0.011)^***^ | 0.457 (0.013)^***^ |
| Depression → Depression | 0.373 (0.011)^***^ | 0.454 (0.013)^***^ |
| IGD → IGD | 0.382 (0.012)^***^ | 0.450 (0.012)^***^ |
| **Cross-lagged paths (hypothesised direction)** |  |  |
| ADHD → Bullying | 0.028 (0.009)^**^ | 0.042 (0.009)^***^ |
| ADHD → Depression | 0.072 (0.009)^***^ | 0.160 (0.011)^***^ |
| ADHD → IGD | 0.051 (0.009)^***^ | 0.079 (0.011)^***^ |
| Bullying → Depression | 0.091 (0.010)^***^ | 0.089 (0.014)^***^ |
| Bullying → IGD | 0.036 (0.010)^***^ | 0.037 (0.011)*** |
| Depression → IGD | 0.060 (0.012)^***^ | 0.054 (0.014)^***^ |
| **Cross-lagged paths (reverse/alternative direction)** |  |  |
| Depression → Bullying | 0.086 (0.011)^***^ | 0.064 (0.010)^***^ |
| IGD → ADHD | 0.041 (0.010)^***^ | 0.056 (0.009)^***^ |
| IGD → Bullying | 0.020 (0.011) | 0.020 (0.011) |
| IGD → Depression | 0.055 (0.010)^***^ | 0.053 (0.011)^***^ |

Note: Values are standardised coefficients with standard errors in parentheses. All models adjusted for baseline covariates. *** p < 0.001; ** p < 0.01; * p < 0.05.

Abbreviations: ADHD, attention-deficit/hyperactivity disorder; IGD, internet gaming disorder.

**Table S5. Indirect effects within the cross-lagged panel model across three waves (standardised estimates)**

| **Indirect pathway** | **Standardised indirect effect** | **95% CI** |
| --- | --- | --- |
| **Indirect effects from ADHD symptoms (Wave 1) to IGD severity (Wave 3)** |  |  |
| ADHD → Bullying → IGD | 0.001 | [0.001, 0.002] |
| ADHD → Depression → IGD | 0.003 | [0.002, 0.005] |
| **Total indirect effect (ADHD → IGD)** | 0.005 | [0.003, 0.007] |
| **Process-level indirect pathways** |  |  |
| ADHD → Bullying → Depression | 0.003 | [0.001, 0.005] |
| Bullying → Depression → IGD | 0.005 | [0.003, 0.008] |

Abbreviations: ADHD, attention-deficit/hyperactivity disorder; CI, confidence interval; IGD, internet gaming disorder.
